# Image-Derived Input Function for Human Brain Using High Resolution PET Imaging with [^11^C](*R*)-rolipram and [^11^C]PBR28

**DOI:** 10.1371/journal.pone.0017056

**Published:** 2011-02-25

**Authors:** Paolo Zanotti-Fregonara, Jeih-San Liow, Masahiro Fujita, Elodie Dusch, Sami S. Zoghbi, Elise Luong, Ronald Boellaard, Victor W. Pike, Claude Comtat, Robert B. Innis

**Affiliations:** 1 Molecular Imaging Branch, National Institute of Mental Health (NIMH), National Institutes of Health (NIH), Bethesda, Maryland, United States of America; 2 CEA/SHFJ, Orsay, France; 3 Department of Nuclear Medicine, VU University Medical Center, Amsterdam, The Netherlands; University of Texas, M.D. Anderson Cancer Center, United States of America

## Abstract

**Background:**

The aim of this study was to test seven previously published image-input methods in state-of-the-art high resolution PET brain images. Images were obtained with a High Resolution Research Tomograph plus a resolution-recovery reconstruction algorithm using two different radioligands with different radiometabolite fractions. Three of the methods required arterial blood samples to scale the image-input, and four were blood-free methods.

**Methods:**

All seven methods were tested on twelve scans with [^11^C](*R*)-rolipram, which has a low radiometabolite fraction, and on nineteen scans with [^11^C]PBR28 (high radiometabolite fraction). Logan *V*_T_ values for both blood and image inputs were calculated using the metabolite-corrected input functions. The agreement of image-derived Logan *V*_T_ values with the reference blood-derived Logan *V*_T_ values was quantified using a scoring system. Using the image input methods that gave the most accurate results with Logan analysis, we also performed kinetic modelling with a two-tissue compartment model.

**Results:**

For both radioligands the highest scores were obtained with two blood-based methods, while the blood-free methods generally performed poorly. All methods gave higher scores with [^11^C](*R*)-rolipram, which has a lower metabolite fraction. Compartment modeling gave less reliable results, especially for the estimation of individual rate constants.

**Conclusion:**

Our study shows that: 1) Image input methods that are validated for a specific tracer and a specific machine may not perform equally well in a different setting; 2) despite the use of high resolution PET images, blood samples are still necessary to obtain a reliable image input function; 3) the accuracy of image input may also vary between radioligands depending on the magnitude of the radiometabolite fraction: the higher the metabolite fraction of a given tracer (e.g., [^11^C]PBR28), the more difficult it is to obtain a reliable image-derived input function; and 4) in association with image inputs, graphical analyses should be preferred over compartmental modelling.

## Introduction

Using radioligands that bind to specific receptors and enzymes, positron emission tomography (PET) can quantify the in vivo density of such targets in brain. This quantification, however, often requires the concurrent measurement of the concentrations of unchanged radioligand in arterial plasma, which is the input function to the brain. Although insertion of an arterial catheter rarely results in clinically relevant adverse events [1], it is laborious and often discourages subjects from volunteering for PET studies. As an alternative to arterial sampling, many methods have been proposed to calculate the input function from serial images of the internal carotid artery – i.e., an image-derived input function [2], [3], [4], [5], [6], [7], [8]. Such methods have been validated for PET cameras with a standard resolution (typically>6 mm). Some of these methods require at least one blood sample in order to scale the image-input, while others are completely blood-free, and therefore more attractive. Unfortunately, blood-free methods seem to be less accurate than blood-based methods when using a PET camera with standard resolution [9], [10], where partial volume effects are more challenging to correct. The accuracy of blood-free methods has yet to be verified using modern high resolution images. High resolution images can be obtained using a tomograph with a higher intrinsic resolution, like the HRRT (High Resolution Research Tomograph; resolution = 2.5 mm), or using reconstruction-based resolution recovery algorithms, which are now implemented on many standard resolution PET machines. These algorithms yield image resolutions comparable to those offered by the HRRT scanner [11].

Furthermore, most of the brain image-derived input methods published in the literature have been validated for [^18^F]FDG. However, neuroreceptor radioligands may differ in some important ways, including their brain distribution, vascular wash-out, and the presence of radiometabolites in blood. As a consequence, the accuracy of a given method may depend on the biokinetics of the radioligand.

The aims of this study were 1) to test seven previously published image-derived input methods (three blood-based [2], [4], [12] and four blood-free methods [5], [6], [8], [13]) from carotid arteries using high resolution PET images, which were obtained using a HRRT and a reconstruction-based resolution recovery algorithm, and 2) to determine whether the percentage of radiometabolites in blood affects quantitation with image input. Thus, we selected two radioligands—[^11^C](*R*)-rolipram and [^11^C]PBR28—with very different percentages of radiometabolites in blood at late scan times. [^11^C](*R*)-rolipram is a probe for the enzyme phosphodiesterase 4 and has a low radiometabolite fraction - i.e. the unchanged parent is the predominant portion of blood radioactivity throughout the scan (about 80% at 90 minutes). [^11^C]PBR28 binds to the translocator protein (18 kDa) and has a high radiometabolite fraction, i.e. the parent radioligand represents about 7% of total blood radioactivity at 90 minutes.

## Materials and Methods

### Subjects

Data from twelve [^11^C](*R*)-rolipram (mean injected activity: 410±119 MBq) and nineteen [^11^C]PBR28 (503±41 MBq) studies were used. Radioligands were injected in bolus over one minute. The [^11^C](*R*)-rolipram subjects were all healthy volunteers, while the [^11^C]PBR28 group comprised seven healthy volunteers and twelve multiple sclerosis patients. [^11^C](*R*)-rolipram is a tracer for phosphodiesterase 4, a selective inhibitor of the cyclic adenosine monophosphate (cAMP) cascade [14]. The cAMP cascade is thought to play important roles in several psychiatric illnesses, including depression [15] and drug addiction [16]. [^11^C]PBR28 is a marker for inflammation. It binds selectively to the translocator protein, a mitochondrial transmembrane protein expressed in activated macrophages and in glial cells [17]. [^11^C](*R*)-rolipram and [^11^C]PBR28 were prepared in greater than 99% radiochemical purity and high specific radioactivity. All scans were acquired in ongoing clinical research studies that had been approved by the Ethics Committee of the NIH. Written informed consent was obtained from each subject.

### PET scans

All scans were acquired using the HRRT (Siemens/CPS, Knoxville, TN, USA) for 120 minutes in 33 frames except one [^11^C](*R*)-rolipram subject who had a 90-minute scan. Because previous studies had shown that distribution volume values are stably estimated within about 90 minutes of image acquisition for both tracers [14], [18], the present study only analyzed the first 90 minutes of the scans for each subject. The dynamic scan consisted of 6 frames of 30 seconds each, then 3×60 s, 2×120 s and 17×300 s. All PET images were corrected for attenuation and scatter [19]. Head motion during each scanning session was corrected by monitoring the position of the head during the scan with the Polaris Vicra Optical Tracking System (NDI, Waterloo, ON, Canada) [20]. During the acquisition, blood samples (1 mL each) were drawn from the radial artery at 15-second intervals until 150 seconds, followed by 3 mL samples at 3, 4, 6, 8, 10, 15, 20, 30, 40, and 50 minutes, and 4.5 mL at 60, 75, 90, and 120 minutes. Whole-blood activity, the fraction of unchanged radioligand in plasma, and the plasma/whole blood ratio were calculated [21], [22].

### Magnetic resonance imaging (MRI)

To identify brain regions, MRIs were obtained for all subjects using a 1.5-T GE Signa scanner (GE Healthcare, Piscataway, NJ, USA). Three sets of axial images were acquired parallel to the anterior-commissure-posterior commissure line with Spoiled Gradient Recalled sequence of TR/TE/flip angle = 12.4 ms/5.3 ms/20°, voxel size = 0.94×0.94×1.2 mm, and matrix = 256×256. These three MRI sets were realigned and averaged, and then coregistered to the PET images (see below).

### Image analysis

PET data were reconstructed on a 256×256 matrix with a pixel size of 1.22×1.22×1.23 mm^3^ using the Motion-compensation OSEM List-mode Algorithm (MOLAR) for Resolution-recovery [19]. Preset volumes of interest drawn in a standard space were applied to each subject's PET images as follows. The average MR image from three acquisitions for each subject (see above) was coregistered using SPM5 to the average PET image created from all frames. Both MR and all PET images were spatially normalized to a standard anatomic orientation (Montreal Neurological Institute space) based on transformation parameters from the MR images. Preset volumes of interest were positioned on the spatially normalized MR images to overlie thalamus (12.6 cm^3^), caudate (5.6 cm^3^), putamen (6.5 cm^3^), cerebellum (51.2 cm^3^), frontal (27.2 cm^3^), parietal (26.6 cm^3^), lateral temporal (25.0 cm^3^), occipital (31.2 cm^3^), anterior cingulate (7.5 cm^3^), and medial temporal (14.3 cm^3^) cortices. Image analysis was performed using PMOD 3.0 (pixel-wise modeling software; PMOD Technologies Ltd., Zurich, Switzerland) and the BrainVISA/Anatomist software (SHFJ/Neurospin, CEA, Orsay, France).

### Input Function Extraction Methods

The seven methods compared in this study can be classified into two groups: the first three are blood-based methods (those which require some blood samples to scale the image-input) and the last four are blood-free methods.

#### Chen's method

Carotid and background regions of interest were manually drawn directly on the summed PET frames of the first two minutes and then copied to all the frames (the same sets of regions of interest (ROI) were also used to calculate the image input using the methods of Su and Parker; see below). The carotid signal measured from the images was represented as a linear combination of the radioactivity from the blood and spill-in from the surrounding tissue [2]:
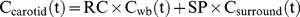
(1)where C_carotid_ is the concentration of radioactivity obtained from the carotid region in the PET image; C_wb_ is the concentration of radioactivity in whole blood sampled directly from the radial catheter; RC is the recovery coefficient necessary to correct the carotid PET measurement for resolution blurring effect; SP is the percentage spill-in from surrounding tissues to the carotid region; and C_surround_ is the radioactivity in the surrounding tissues obtained from a comma-shaped ROI drawn in the proximity, but not immediately adjacent, to the carotid region. RC and SP were estimated with the linear least squares method by using C_carotid_, C_wb_, and C_surround_ in equation (1) at 6, 20, 60 and 90 minutes for [^11^C](*R*)-rolipram studies and at 4, 20, 60 and 90 minutes for [^11^C]PBR28 studies.

#### Mourik's method

This approach takes advantage of a reconstruction algorithm that includes modelling of the tomograph spatial resolution [12]. During reconstruction with the Motion-compensation OSEM List-mode Algorithm, resolution recovery is achieved by the use of a 3D Gaussian function with full width at half maximum (FWHM) of 2.5 mm [19]. The image-input was obtained by averaging the cluster of the four hottest pixels per plane within the carotids, as defined in the early summed frames. The resulting image-input curves were first fitted with a tri-exponential function, and then scaled using the mean of the ratio between three arterial whole blood samples at 20, 60, and 90 minutes, and the activity of the fitted image-input at the corresponding time points.

#### Naganawa's method

The approach proposed by Naganawa and colleagues is based on the Independent Component Analysis (ICA) [4]. With ICA, time-activity curves are extracted without any anatomical assumption and spill-over effects are implicitly accounted for through the source signal mixing process. The ICA algorithm used in the present study is EPICA (http://home.att.ne.jp/lemon/mikan/EPICA.html). Images were first cropped in order to eliminate most of the background activity and then smoothed with a 6 mm FWHM Gaussian filter (smoothing was necessary in order to better identify image-input, owing to the sensitivity of the ICA algorithm to the noise of HRRT images). Because ICA cannot determine the absolute amplitude of the independent components, we scaled the final curves using Mourik's three-blood-sample method described above.

#### Su's method

Su used Chen's formula, but replaced the whole-blood sample values with the local frame-wise hottest voxel from the carotids over the first 30 minutes of the acquisition after [^18^F]FDG injection [5]. In order to assess the optimal time for our tracers, we increasingly truncated, with 10 minute increments, the hottest voxel time-activity curves from the initial length of 90 minutes to only the initial 10 minutes and calculated the corresponding image input function at every step. The mean area under the curve for each set of image inputs was compared to the mean area under the curve of the reference arterial input. Timing that gave the ratio closest to 1 was selected. The final *V*_T_ values were calculated from image-inputs obtained using the hottest carotid voxel for the first 20 minutes for [^11^C](*R*)-rolipram and the first 40 minutes for [^11^C]PBR28.

#### Parker's method

This method also relies on Chen's basic formula. The whole-blood samples values are estimated by using I_max_, which is derived from the mean of the hottest 5% data points in the carotids [6]. At the end of the scan, if the surrounding tissue activity exceeds blood activity, I_max_ is corrected to I_max_×I_mean_/T_mean_, where I_mean_ and T_mean_ are the mean values over the carotid and over the tissue background regions, respectively.

#### Backes' method

The whole-blood concentration, C_wb_(t), is obtained from the measured concentration C_carotid_(t) by:
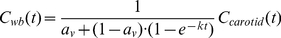
(2)where *a_v_* takes into account the fractional volume of the vessel within the ROI, and *k* is the constant for the transport from the vessel to the surrounding tissue [13]. The factors *a_v_* and *k* must be empirically determined. To extract carotid time-activity curves, we used two squared ROIs centered on each carotid over four slices. We applied equation (2) using combinations of *a_v_* values of 0.3, 0.4, and 0.5, and *k* values of 5×10^−1^, 5×10^−2^, 5×10^−3^, and 5×10^−4^ min^−1^ to calculate the image input. We then compared the mean area under the curve for each set of image inputs to the mean area under the curve of the reference arterial input and selected the combination of values that gave the ratio closest to 1. The chosen values were *a_v_* = 0.4 and *k* = 5×10^−4^ min^−1^ for [^11^C](*R*)-rolipram, and *a_v_* = 0.5 and *k* = 5×10^−2^ min^−1^ for [^11^C]PBR28 studies.

#### Croteau's method

The carotid diameter was measured on the MRI scan of each subject by averaging the diameters of both carotids over three adjacent planes below the skull. In the same planes, carotid time-activity curves were obtained by averaging the four hottest pixels on the PET images. The final, very noisy, time-activity curves were first fitted with a tri-exponential function and then corrected for partial volume effect (but not for spill-in) by applying a recovery coefficient proportional to the carotid diameter [8]. Recovery coefficients were obtained by averaging the four hottest pixels of the images obtained from an analytic simulator, using the same resolution and voxel size of the PET images. Recovery coefficients were calculated for diameters ranging from 3 to 8 mm, with 0.1 mm steps. To test the accuracy of the simulated coefficients, we imaged 2 syringes on the HRRT, with a diameter of 3.6 and 4.8 mm respectively, containing a concentration of 2.9 MBq/mL of ^11^C. The mean simulated/measured coefficient ratio for these two diameters was 0.995. In contrast to Croteau's paper, we did not calculate the relationship between the FWHM of the object measured on the PET images and the actual diameter of the object. Instead, we directly used the carotid diameter measured on the MRI as a more precise way to determine carotid size, and hence the correct recovery coefficient.

### Figures of merit

#### Visual comparison

Whole-blood image-inputs obtained with each method were first visually compared with the reference arterial whole-blood time-activity curves. We compared the overall shape of the curves and in particular how well the image inputs matched the height of the peaks and the slope of the tails of the reference arterial curves.

#### Area under the curve (AUC) ratios

The mean area under the curve (AUC) ratio between the image-derived and arterial time-activity curves was calculated for each tracer. We compared the image/arterial AUC ratios of both the whole-blood and the metabolite-corrected parent time-activity curves. Metabolite correction for the image-inputs was performed by multiplying the image-derived whole blood curve with the parent/whole blood ratios at each time point (interpolated to agree with the scanner time points at the beginning of each frame).

#### Kinetic modelling

Both arterial and image inputs were first corrected for metabolites, then distribution volume (*V*_T_) values were obtained using the Logan analysis. We calculated the image/blood mean Logan *V*_T_ ratio for each subject. A scoring system was used to compare the different methods. We gave 2 points if the image/arterial *V*_T_ ratio comprised between ±5%, 1 point if comprised between ±5–10%, and 0 points if higher than ±10%. Compartment modeling was also performed using the methods that gave the most accurate results with Logan analysis. Delay of the input functions was corrected by fitting the input functions with the brain time-activity curves. The blood volume was set at 0 and *V*_T_ values and individual rate constants (K_1_, k_2_, k_3_ and k_4_) were calculated using an unconstrained two-tissue compartment model. We calculated the image/blood mean ratio of these parameters for each subject. When the non-linear least square fitting occasionally did not converge in a parameter of one region, that region was excluded from the analysis.

### Phantom simulations

We used two MRI-based numerical phantoms of the human brain [23], into which two sets of internal carotids, with a diameter of 8 and 5 mm respectively, were added. The phantoms consisted of 19 anatomic labels, corresponding to the anatomical regions of the head of the phantoms, including carotids, frontal, temporal, parietal and occipital grey matter, white matter, basal ganglia, bones, and soft tissues. For each label, we defined a time-activity curve, obtained by averaging the corresponding time-activity curves of the [^11^C](*R*)-rolipram and [^11^C]PBR28 clinical studies. The time-activity curve defined for carotid labels was the reference “arterial” whole-blood input function. The simulated time-activity curves for each region were used as input of an analytic fast simulator [24], recently upgraded to simulate HRRT studies [25]. This simulator takes into account true, scattered, and random coincidence detector efficiencies and includes a realistic detector resolution model for the HRRT [25]. Noisy sinograms were generated based on the same time frame used in the clinical studies. A realistic noise level was achieved by calibrating the true, random, and scatter events and noise-equivalent count rates of the phantoms with those of the clinical data. The simulated noisy sinograms were reconstructed with the OP-OSEM algorithm, with 16 subsets and 10 iterations. The voxel size of the reconstructed images was set to 1.22×1.22×1.22 mm^3^. The image-based Point Spread Function model was used during the reconstruction, in both the forward- and back-projection, with an isotropic and stationary 3D kernel given by:
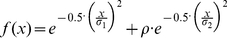
(4)With σ_1_ = 0.9 mm, σ_2_ = 2.5 mm, and ρ = 0.07. This resolution model was validated for the HRRT scanner by Comtat and colleagues [26].

**Figure 1:**
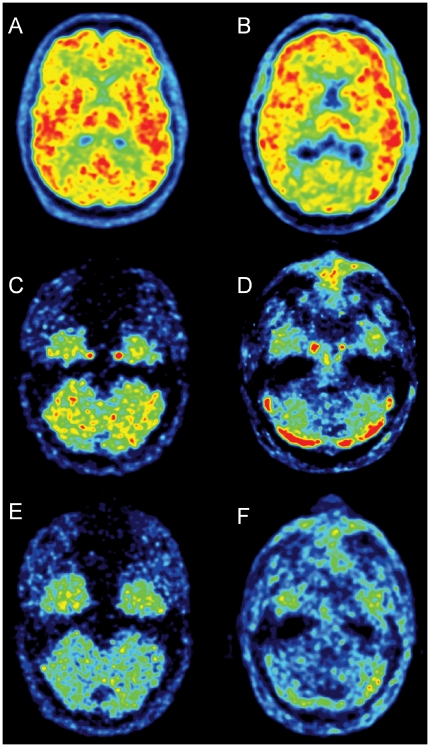
Transaxial slices from a [^11^C](*R*)-rolipram brain scan of a healthy volunteer and from a simulated study using a digital phantom. Upper row: [^11^C](*R*)-rolipram images across the thalamus summed over the whole duration of the scan from a phantom (**A**) and a healthy volunteer (**B**). The phantom images are realistic and quite similar to those from the real subjects. The external rim of activity surrounding the brain, in both the subject and the phantom, is scalp activity. Middle row: images summed over the first two minutes at the carotid level. The carotids are well visible near the temporal lobes for both the phantom (**C**) and the healthy volunteer (**D**). The regions of high activity visible in the lower part of the cerebellum of the subject (**D**) are the cerebellar venous sinuses (not simulated in the phantom studies). Bottom row: late images (three summed frames taken at about 1 hour after injection) from a phantom (**E**) and a subject (**F**). At late times the carotids are not well visible anymore and the spill-over effect from surrounding tissues becomes more important.

In total, the dynamic PET phantom was computed by linear combination of the phantom structures, weighted by the associated kinetics, and sampled into time frames whose number and duration time were identical to those of the clinical studies (see below). The dynamic phantom was forward projected and noise was added, taking into account scattered and random coincidences (Figure 1). Image-input was calculated for all phantoms using the methods of Chen and Mourik. The fraction of unchanged parent was derived by multiplying the “arterial” and image input of the phantoms by the average parent/whole blood time activity curve measured in the clinical scans, after linear interpolation of the blood data to match the PET time schedule.

## Results

### Blood analyses

**Figure 2:**
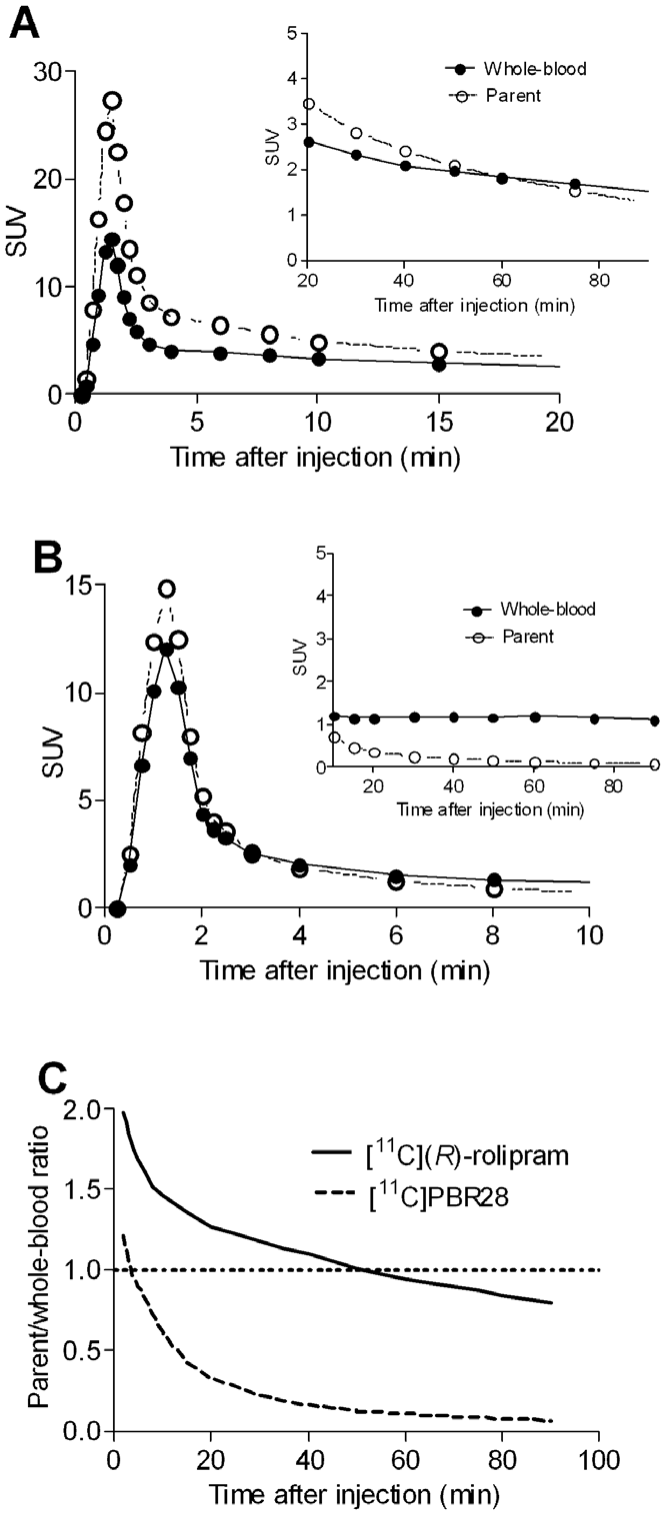
The average concentrations of radioactivity in whole blood (solid line) and parent radioligand in plasma (dashed line) over time for [^11^C](*R*)-rolipram (n = 12) (A) and [^11^C]PBR28 (n = 19) (B). The main figures show the first 20 minutes of the curves and the inserts the remaining part. Although the shape of the whole blood curves was similar for the two tracers, the relative concentration of parent and metabolites differed. The mean ratio of concentration of parent radioligand in plasma to total radioactivity in whole blood (**C**) showed that [^11^C](*R*)-rolipram remained the predominant component of whole blood radioactivity throughout the scan. In contrast, radiometabolites of [^11^C]PBR28 became the predominant component of whole blood radioactivity after the first few minutes.

The shape of the whole-blood curves was very similar for the two tracers (Figure 2AB), with a concentration peak at ∼90 seconds and a rapid decline thereafter; however, the relative concentration of parent and metabolites differed (Figure 2C). [^11^C](*R*)-rolipram remained the predominant portion of blood radioactivity throughout the scan. The mean parent/whole blood ratio was of about 1 (0.99±0.24) at 60 minutes after injection, and 0.80±0.30 at 90 minutes. In contrast, for [^11^C]PBR28, radiometabolites became the predominant component of blood radioactivity for most of the scan. The mean parent/whole blood ratio was of about 1 (0.96±0.13) at 4 minutes after injection, and 0.07±0.02 at 90 minutes. The mean/whole blood ratios are calculated from all the subjects used in this study (n = 12 for [^11^C](*R*)-rolipram and n = 19 for [^11^C]PBR28).

### Accuracy of the image-derived input function

#### Visual analysis

**Figure 3:**
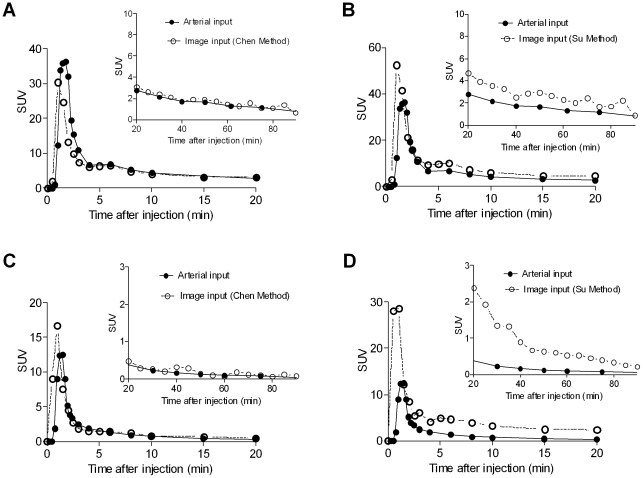
The concentrations over time of [^11^C](*R*)-rolipram (A and B) and [^11^C]PBR28 (C and D) in plasma from the arterial input function (solid line) and from the image input function (dashed line) of a representative healthy subject. The curves are representative of those from a blood-based (Chen; **A and C**) and a blood-free (Su; **B and D**). None of the methods precisely estimated the peak in all the subjects but, in general, blood-based methods yielded a better estimate of the tails of the curves.

For both tracers, none of the methods could consistently reproduce the height and shape of the reference arterial peaks. In general, however, the blood-based methods provided a better estimate of the late part (i.e. the tails) of the curves than the blood-free methods (Figure 3).

Using Chen's methods, the tails, but not the peaks, of image-inputs matched closely the arterial inputs for both tracers. The peaks were generally slightly underestimated, although an overestimation was observed for several patients. Mourik's method gave similar results: a closely matching tail and a less accurate peak, although with this method an overestimation of the peak was more common. With Naganawa's method the late part of the tail of the image-inputs, in general, successfully followed the reference arterial input. However, there were often considerable errors in the estimation of the peaks (and often in the early part of the tails), both in the height (significant under- and over-estimations) and in the shape (sometimes a bicuspid peak was observed). Using the methods of Su and Parker, overestimations of both the peak and the tail of the curves, to a variable degree, were observed for most subjects and for both tracers. With the method of Backes, the peaks were consistently underestimated for all subjects and for both tracers, while the tails sometimes showed an under- or over-estimation, but usually of modest entity. Finally, Croteau's method underestimated the curves to a variable degree with both tracers.

#### Area under the curve ratios

In addition to the visual analysis, we performed a quantitative analysis by calculating the mean ratio of the AUCs for both the whole-blood and parent curves. In general, the difference of the mean AUC ratio was smaller for the blood-based methods than for the blood-free methods. Interestingly, while no significant difference was noted between the whole-blood and parent AUC ratios for [^11^C](*R*)-rolipram, the whole-blood AUC ratios for [^11^C]PBR28 were on average better than the parent ratios (Table 1).

**Table 1:** AUC ratio (mean ± SD) calculated for each method and for both whole-blood and plasma curves for each tracer.

		Blood-based methods			Blood-free methods			
		Chen	Mourik	Naganawa	Su	Parker	Backes	Croteau
[^11^C](*R*)-rolipram	Whole-blood	1.03±0.04	1.07±0.10	0.96±0.21	1.49±0.28	1.28±0.21	1.03±0.10	0.36±0.12
	Plasma	1.00±0.04	1.06±0.13	0.98±0.38	1.47±0.26	1.24±0.20	0.98±0.09	0.35±0.14
[^11^C]PBR28	Whole-blood	1.03±0.12	0.96±0.04	0.96±0.08	3.69±1.57	2.65±0.76	1.00±0.24	0.55±0.11
	Plasma	0.93±0.15	0.92±0.09	0.91±0.19	3.07±1.24	2.18±0.60	0.85±0.21	0.52±0.10

The AUC ratio is on average more accurate for blood-based methods than for blood-free methods. For [^11^C]PBR28, but not for [^11^C](*R*)-rolipram, the parent AUC ratios of the blood-based methods are less accurate than the whole-blood AUC ratios.

For [^11^C](*R*)-rolipram, the difference of mean AUC ratio was <10% for the three blood-based methods and for the method of Backes in the whole-blood curves; these values did not significantly change after metabolite correction. For the methods of Su and Parker the mean ratio was much higher than 1 for both sets of curves, while Croteau's method yielded a ratio much lower than 1 (Table 1).

For [^11^C]PBR28, the difference of the mean AUC ratio for the whole blood curve was <10% for the three blood-based methods and for the method of Backes. After metabolite correction, the AUC ratio for the parent time-activity curves showed a greater error for all these four methods. Much less accurate results were obtained for both sets of curves with the three remaining blood-free methods (Table 1).

#### Kinetic modelling

As expected from the results of the AUC ratios, the best results were obtained for both tracers with two of the blood-based methods—those of Chen (22/24 for [^11^C](*R*)-rolipram) and Mourik (18/38 for [^11^C]PBR28). The best results using a blood-free method were obtained using the method of Backes (13/24 for [^11^C](*R*)-rolipram and 9/38 for [^11^C]PBR28). Notably, the scores for each method were consistently higher for [^11^C](*R*)-rolipram than for [^11^C]PBR28 (Table 2). When the image input gave an inaccurate estimation of the reference input function, the error in *V*_T_ estimation was of the same magnitude in all the brain regions, regardless of the respective binding levels.

**Table 2:** Image/blood *V*_T_ ratios (mean ± SD) and scores calculated for each method.

		Blood-based methods					Blood-free methods			
		Chen (Logan)	Chen (2TCM)	Mourik (Logan)	Mourik (2TCM)	Naganawa (Logan)	Su (Logan)	Parker (Logan)	Backes (Logan)	Croteau (Logan)
[^11^C](*R*)-rolipram	*V*_T_ Ratio	0.99±0.04	1.00±0.05	0.97±0.07	1.22±0.32	1.09±0.19	0.74±0.19	0.82±0.15	0.97±0.11	3.35±1.81
	Score	22/24	19/24	16/24	7/24	6/24	3/24	4/24	13/24	0/24
[^11^C]PBR28	*V*_T_ Ratio	1.10±0.17	1.15±0.20	1.08±0.09	1.29±0.23	1.11±0.14	0.36±0.14	0.46±0.11	1.19±0.28	1.94±0.43
	Score	11/38	12/38	18/38	5/38	7/38	0/38	0/38	9/38	0/38

The scores are calculated by giving 2 points each time the image/arterial *V*_T_ ratio comprised between ±5%, 1 point if comprised between ±5–10%, and 0 points if higher than ±10%. The most accurate results for both tracers were obtained using two blood-based methods (Chen and Mourik) and the Logan plot. When *V*_T_ ratios were calculated using these two blood-based methods and an unconstrained two-tissue compartment model (2TCM), the overall results were less accurate. Please note that even if the Chen-[^11^C]PBR28 score is comparable between the two modeling approaches (11/38 vs. 12/38), the 2TCM yields a greater bias and a greater standard deviation of the mean *V*_T_ ratio (1.10±0.17 vs. 1.15±0.20).

Compartment modeling was performed for both tracers using the image inputs obtained with the methods of Chen and Mourik and an unconstrained two-tissue compartment model. The resulting individual rate constants showed important and unpredictable errors in both tracers and with both methods (Table 3). Consequently, the *V*_T_ values obtained from compartment modeling, which are derived from the individual rate constants, were much less accurate than those obtained with the Logan plots. For example, the score for [^11^C](*R*)-rolipram changed from 22/24 to 19/24 using the method of Chen and from 16/24 to 7/24 using the method of Mourik (Table 2).

**Table 3:** Image/blood ratio of the individual rate constants obtained with an unconstrained two-tissue compartment model.

	Chen				Mourik			
	K_1_	k_2_	k_3_	k_4_	K_1_	k_2_	k_3_	k_4_
[^11^C](*R*)-rolipram	1.78±0.29	2.07±2.42	1.30±1.16	1.13±0.50	0.82±0.29	0.81±0.43	1.09±0.89	0.85±0.58
[^11^C]PBR28	1.96±1.40	1.64±1.27	0.80±0.29	0.91±0.42	1.18±0.33	0.71±0.30	0.46±0.21	0.68±0.18

### Phantom simulations

We suspected that an imperfect estimation of the peak, due to a coarse temporal framing, was responsible for the more accurate Logan *V*_T_ estimations with [^11^C](*R*)-rolipram than with [^11^C]PBR28. To verify this hypothesis, we performed digital phantom simulations. In these phantoms, the carotid signal is not averaged over the duration of the frames, because each frame corresponds to a punctual detection at a given time. All the other possible sources of error in the estimation of the peaks (i.e. noise, random counts, partial volume effect, spill-over) are faithfully simulated. Our results show that, when the peak signal is not averaged over the duration of the frames, image-input methods may accurately estimate Logan *V*_T_ values even for tracers with a high metabolite fraction (like [^11^C]PBR28). Indeed, a correct estimation of the peak is more important for [^11^C]PBR28 than for [^11^C](*R*)-rolipram (see Discussion).

The image-inputs estimated with the methods of Chen and Mourik were very similar to the reference input functions for the four phantom simulations. The tails of the curves matched closely, and only minor differences in height were observed for the peaks. For both tracers, the mean AUC ratio was close to 1, both before and after metabolite correction. No significant differences were noted between the curves calculated from carotids of different size. The Logan *V*_T_ estimations were very accurate for both [^11^C](*R*)-rolipram and [^11^C]PBR28 phantoms (Table 4). For [^11^C](*R*)-rolipram, the resulting Logan *V*_T_ values were similar to those found in the clinical scans, while for [^11^C]PBR28 the phantom Logan *V*_T_ values were more accurate than the average values obtained from the clinical data.

**Table 4:** Results of the phantom simulation.

	[^11^C](*R*)-rolipram		[^11^C]-PBR28	
	Chen	Mourik	Chen	Mourik
Whole-blood AUC ratio	1.059	1.007	1.019	1.025
Plasma AUC ratio	1.049	1.007	1.027	1.068
Logan *V*_T_ ratio	0.948	0.994	0.986	0.949

## Discussion

This study tested seven different image-derived input function methods on dynamic high resolution brain PET scans. Three of these methods require blood samples to scale the image input and four are blood-free. Corroborating a previous study done on standard resolution PET images [9], we found that the most accurate image input estimations were obtained using two blood-based image input methods (Chen and Mourik), while blood-free methods were generally less reliable. Chen's method uses some blood samples to estimate the partial volume and spill-over correction coefficient, which results in good estimates of the tail of the input function [2]. Mourik's method relies on defining small ROIs within the carotids to avoid contamination by the background activity. This method allows the estimation of a completely blood-free image input with some tracers on PET machines with a standard resolution [3], [27]. However, blood samples are necessary when using this method on HRRT, probably because of a higher scatter contribution [12]. Mourik's method also provides a better estimate of the peak than the other methods assessed here, which is particularly important for tracers with a high metabolite fraction (see below). Naganawa's method takes advantage of ICA to extract the image-input without any anatomical prior. This clever method has been shown to yield good results with clinical PET scanners for [^18^F]FDG [4] and [^11^C]MPDX, a tracer for adenosine receptors [28]. However, in the present study, results with this method were less accurate, likely owing to the sensitivity of ICA to noise associated with high resolution images.

Performance of the four blood-free methods was generally poor. The methods of Su and Parker yielded an inaccurate estimate of *V*_T_ values with both tracers, mainly because these two methods gave a poor estimation not only of the peaks, but also of the tails of the input functions. In general, blood-free methods that rely on a limited number of voxels to estimate blood activity are theoretically attractive. However, their accuracy may be heavily and unpredictably influenced by a number of parameters such as noise levels, scatter correction methods, reconstruction algorithms, filtering parameters, and tracer biodistribution. Among the blood-free methods assessed here, the best results were obtained with the method of Backes. This method is less sensitive to noise, and the parameters *a_v_* and *k* are independent of scanner type [13]. Because this method was originally validated on a standard resolution PET machine using venous sinuses as a source of image-derived input, the carotid blood pool should theoretically provide a more accurate estimate of the input function. However, Backes showed that because of the small size and sensitivity to motion, the carotid time-activity curves were too noisy to be used for kinetic modeling [13]. In the present study, images had a higher spatial resolution and movements were corrected by an on-line motion correction system. Therefore, the inaccurate results sometimes found with this method are probably due to inter-subject variability in carotid size and in the tracer diffusion to the extravascular compartment, i.e. the *a_v_* and *k* factors of the formula (2). Such inter-subject variability is not taken into account in (2).

Croteau's method yielded poor results with both tracers. This method seems to be very sensitive to errors. Croteau showed that an underestimation of the diameter of the carotid artery by just 1 mm would induce an error in the cerebral metabolic rate of glucose of about 17% [8]. Even larger errors were found when this method was applied to femoral arteries: an under/overestimation of the artery size of 1 mm entailed an under/overestimation of ∼66% in the perfusion index measured with [^11^C]acetate [8]. Clearly, the scaling of the image input through recovery coefficients can be very sensitive to errors, and scaling with blood samples should be preferred.

In summary, most of the image input methods tested in the present study on [^11^C](*R*)-rolipram and [^11^C]PBR28 gave poor results. This suggests that image input methods that are validated and work well on a given tracer do not necessarily perform equally well when applied to other tracers (despite the use of high resolution images). For instance, using the image input method we previously validated for [^11^C](*R*)-rolipram [14], we were unable to obtain equally reliable results for any other tracer of our database. That is because different tracers may show different biodistribution characteristics, such as a more or less strong carotid signal or different levels of background (i.e. cortical and soft tissue) uptake. As a consequence, a different biodistribution may entail spill-over effects of different magnitude. In our (unpublished) experience, among the factors that make image input methods fail there are a low carotid signal and an excessively high early spill-over from surrounding tissues. Examples of such tracers are [^18^F]-FMPEP-d_2_ [29], [^11^C]-MePPEP [30], [^11^C]-DASB [31] and [^18^F]-SP203 [32].

Three of the methods we evaluated in our previous comparison [9] have not been reassessed in the present work. The method of Litton [33] uses recovery coefficients empirically determined to correct for partial volume effect and therefore is similar to the more recent and better validated method of Croteau [8]. The method of Bodvarsson uses a Nonnegative Matrix Factorization approach to extract the input function [34]. However, the factorization algorithm used [35] suffers from the existence of local minima. The use of random matrix to initialize the algorithm can make the algorithm converge to these minima, in particular in noisy high-resolution HRRT images. The third one is a method we previously proposed in abstract form [36], in which partial volume effect is corrected using the Geometric Transfer Matrix approach [37]. However, our method performed poorly when we compared it to other published methods [9]. Subsequent (unpublished) phantom simulations showed that our method does not allow a full recovery of partial volume effect and moreover is very sensitive to minor errors in carotid segmentation. Therefore it is too unreliable to be used in clinical practice.

When a Logan plot is used to obtain *V*_T_, the relationship between the plasma AUC and *V*_T_ values is quite straightforward, as the Logan plot mainly relies on the integral of the AUC. Therefore, an overestimation of about 20% of the plasma AUC would lead to an underestimation of Logan-*V*_T_ of the same order of magnitude. This can be easily seen by comparing the results reported in the plasma AUC (Table 1) to those reported in the *V*_T_ (Table 2). Although the graphical methods may carry forward any error that occurred at the beginning of the curve, since they use progressive integrals of the AUC, in the case of Logan plots this does not matter that much because this technique relies mostly on the late parts of the curve (pseudoequilibrium). In contrast, in the case of Patlak plots any error in the early part of the input function may affect overall scaling and thus have a direct effect on the slope. However, even for Patlak plots, errors in the estimation of the peak should have only moderate consequences on the results of kinetic modeling. Using [^18^F]FDG and the Patlak plot, Chen showed that an underestimation of about 20% of the peak would cause less than 0.1% variation on the estimated metabolic rates of glucose [2].

On the other hand, when using a two-tissue compartmental model, the shape of the input function becomes a critical factor. In fact, *V*_T_ it is derived from the individual rate constants according to the formula 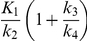. Individual rate constants are very sensitive to variations in the shape of the curve, which may not be well-estimated using image inputs. Corroborating a previous study with [^18^F]-FDG [9], the present study shows that image input does not reliably estimate individual rate constants. Such rate constants will usually carry much larger random errors than compound parameters (such as K_i_, *V*_T_, and Binding Potential). When calculating compound parameters from individual rate constants, some of the random errors in the latter will cancel out, providing relative stability to the compound parameters. However, *V*_T_ calculated with a two-tissue compartmental model was still less accurate than that obtained with the Logan plot. Therefore, the present study and the previous one [9] suggest that image inputs should be preferably used in association with graphical analyses. While producing smaller random errors, graphical methods are however potentially vulnerable to bias and this bias is mostly linked to the accuracy of the estimation of the later parts of the input functions. Therefore, the most reliable results were obtained by those image-input methods that provided a better estimation of the tail of the curves.

One important limitation of image-derived input function is that image inputs cannot distinguish the parent compound from its radioactive metabolites. The different image input methods for neuroreceptor tracers described heretofore in the literature have not addressed the problem of individual metabolite correction. Some studies estimated only the whole-blood curve from images, and the percentage of unchanged parent (the true input function) was obtained at each time point by correcting the image input using HPLC analysis from arterial blood sampling [3], [27], thus diminishing the practical utility of the proposed method. Other studies did not perform metabolite correction [28], [33]. Naganawa and colleagues calculated [^11^C]MPDX whole-blood time-activity curve using ICA and stated that the metabolite fraction for this tracer is negligible [28]. However, according to a previous study, 76% of administered [^11^C]MPDX remains intact 60 minutes after injection [38]. It is questionable whether an error of up to ∼25% in the estimated parent concentration can be safely overlooked.

The use of a population-based average metabolite fraction would eliminate the need of arterial sampling to correct the whole-blood time activity curves obtained from images. However, this approach must be validated for each tracer. In this study, using an average metabolite curve instead of individual metabolite correction for the [^11^C](*R*)-rolipram image inputs calculated with the method of Chen would have caused an important loss of accuracy. In fact, we recalculated the Logan *V_T_* values and the relative scores after metabolite correction using an average population-based metabolite curve. As compared to individual metabolite correction, the mean Logan *V_T_* ratio changed from 0.99±0.04 to 0.98±0.20 and the score changed from 22/24 to only 5/24. A previous study from our laboratory demonstrated that individual metabolite correction can be successfully integrated in the image input calculation algorithm without increasing the invasiveness of the procedure [14]. However, investigating possible approaches of metabolite correction is outside the scope of the present comparative study. Therefore, we performed metabolite correction using the reference method, i.e. calculating the unchanged parent at each time point using HPLC analysis. In this way, we also avoided the additional source of uncertainty associated with estimating the metabolite fraction.

In the present study, we also showed that the magnitude of the metabolite fraction may significantly impact the accuracy of the image-input, as the scores for each method were consistently higher for [^11^C](*R*)-rolipram—which has a lower metabolite fraction in plasma—than for [^11^C]PBR28 scans. The shape of the early part of an input function is characterized by rapid changes in radioactivity concentration over time, and is therefore always difficult to estimate accurately from PET images. The Logan plot uses the AUC of the input function and therefore is not very sensitive to the accuracy of peak estimation. In fact, when we used Chen's method in the present study, we found that the [^11^C](*R*)-rolipram mean image/blood AUC ratio for whole-blood curves was close to 1, and that this figure did not change significantly after metabolite correction (Table 1). Therefore, correctly estimating the peak does not appear to be critical for Logan *V*_T_ calculation in ligands with a low metabolite fraction.

The situation is different in ligands with a high metabolite fraction. For [^11^C]PBR28, after whole-blood curves were corrected for metabolites, the total area under the tail dramatically decreased (Figure 2B), and the accuracy of Logan *V*_T_ values became more dependent on the unreliable area under the peak. While the whole-blood AUC ratio calculated using Chen's method is also close to 1, the mean metabolite-corrected parent AUC ratio is less precise (Table 1). The same pattern is found for all the other methods that provide a good estimation of the tail (Mourik, Naganawa, Backes). This suggests that accurately estimating the peak becomes more critical for ligands with a high metabolite fraction, since the peak now accounts for a larger proportion of the total parent AUC.

Theoretically, a more rapid PET framing should allow for better definition of the peak. However, in short dynamic HRRT frames the noise increases considerably and quantification becomes difficult. Moreover, even using the two most reliable methods (Chen and Mourik), the errors in the peaks estimation were very variable for the same original time-framing. We observed unpredictable underestimations and occasional overestimations of the peaks. This is partly due to the noise in the images, but also raises the hypothesis of possible inaccuracies in the calculation of the reference arterial peaks, which are obtained by discrete arterial sampling. Therefore, to eliminate these confounding factors, instead of modifying the time-framing of the clinical PET scans, we chose to perform phantom simulations, where the height of the peaks is perfectly known.

These simulations proved that the inconsistency of the peak estimation was the principal cause of Logan *V*_T_ inaccuracy using tracers with a high metabolite fraction. In the phantom data, the height of the peaks was perfectly known and the carotid signal was not averaged over the duration of the frames, as each frame corresponded to a punctual detection at a given time; that is, the temporal sampling was “ideal” or “perfectly matched”. All other possible sources of error in estimating the peaks (i.e. noise, random counts, partial volume effect, spill-over) were faithfully simulated. In other words, and contrary to clinical data, the phantom peaks were no more difficult to estimate than the tail. The results of the simulation confirms that a better estimation of the peak is critical for accurately estimating Logan *V*_T_ using tracers with a high metabolite fraction like [^11^C]PBR28. Indeed, a better estimation of the peak has little influence on estimating Logan *V*_T_ values for tracers with a low metabolite fraction, like [^11^C](*R*)-rolipram.

These findings can be extrapolated to other neuroreceptor tracers: the higher the metabolite fraction of a given tracer, the more difficult it will be to obtain a reliable image-derived input function. Therefore, if a tracer is known to have a high metabolite fraction, serial blood sampling is likely to give better results than image input.

In conclusion, our study on image input function of [^11^C](*R*)-rolipram and [^11^C]PBR28 in high resolution PET images has demonstrated that image derived input with limited blood samples works satisfactorily with [^11^C](R)-rolipram but not with [^11^C]PBR28 and when a Logan analysis is used to calculate *V*_T_, but not a two-tissue compartment model. The biokinetics of the two tracers we used in the present study is representative of that of many other tracers. Thus several more generalizable conclusions can be made as follows:

1. Image input methods validated for a specific tracer and a specific machine may not perform equally well in a different setting. Therefore, careful evaluation and previous validation is necessary when applying a method to a particular radioligand in clinical practice.
2. Despite the use of high resolution PET images, blood samples are still necessary for obtaining reliable image input function.
3. The accuracy of image input may also vary between radioligands depending on the magnitude of the radiometabolite fraction: the higher the metabolite fraction of a given tracer, the more difficult it is to obtain a reliable image-derived input function.
4. When using an image input, the total area under the curve of the input function is easier to estimate than its shape. Therefore, kinetic modeling performed using graphical analyses (such as the Logan plot), which rely on the integral of the area under the curve, is likely to give more reliable results than when using compartmental modeling.

## References

[1] Everett BA, Oquendo MA, Abi-Dargham A, Nobler MS, Devanand DP (2009). Safety of radial arterial catheterization in PET research subjects.. J Nucl Med.

[2] Chen K, Bandy D, Reiman E, Huang SC, Lawson M (1998). Noninvasive quantification of the cerebral metabolic rate for glucose using positron emission tomography, 18F-fluoro-2-deoxyglucose, the Patlak method, and an image-derived input function.. J Cereb Blood Flow Metab.

[3] Mourik JE, Lubberink M, Klumpers UM, Comans EF, Lammertsma AA (2008). Partial volume corrected image derived input functions for dynamic PET brain studies: methodology and validation for [11C]flumazenil.. Neuroimage.

[4] Naganawa M, Kimura Y, Ishii K, Oda K, Ishiwata K (2005). Extraction of a plasma time-activity curve from dynamic brain PET images based on independent component analysis.. IEEE Trans Biomed Eng.

[5] Su KH, Wu LC, Liu RS, Wang SJ, Chen JC (2005). Quantification method in [18F]fluorodeoxyglucose brain positron emission tomography using independent component analysis.. Nucl Med Commun.

[6] Parker BJ, Feng DG (2005). Graph-based Mumford-Shah segmentation of dynamic PET with application to input function estimation.. IEEE TRANSACTIONS ON NUCLEAR SCIENCE.

[7] Zanotti-Fregonara P, Maroy R, Comtat C, Jan S, Gaura V (2009). Comparison of 3 Methods of Automated Internal Carotid Segmentation in Human Brain PET Studies: Application to the Estimation of Arterial Input Function.. Journal of Nuclear Medicine.

[8] Croteau E, Lavallee E, Labbe SM, Hubert L, Pifferi F (2010). Image-derived input function in dynamic human PET/CT: methodology and validation with (11)C-acetate and (18)F-fluorothioheptadecanoic acid in muscle and (18)F-fluorodeoxyglucose in brain.. Eur J Nucl Med Mol Imaging.

[9] Zanotti-Fregonara P, Fadaili el M, Maroy R, Comtat C, Souloumiac A (2009). Comparison of eight methods for the estimation of the image-derived input function in dynamic [(18)F]-FDG PET human brain studies.. J Cereb Blood Flow Metab.

[10] Chen K, Chen X, Renaut R, Alexander GE, Bandy D (2007). Characterization of the image-derived carotid artery input function using independent component analysis for the quantitation of [18F] fluorodeoxyglucose positron emission tomography images.. Phys Med Biol.

[11] Mourik JE, Lubberink M, van Velden FH, Kloet RW, van Berckel BN (2010). In vivo validation of reconstruction-based resolution recovery for human brain studies.. J Cereb Blood Flow Metab.

[12] Mourik JE, van Velden FH, Lubberink M, Kloet RW, van Berckel BN (2008). Image derived input functions for dynamic High Resolution Research Tomograph PET brain studies.. Neuroimage.

[13] Backes H, Ullrich R, Neumaier B, Kracht L, Wienhard K (2009). Noninvasive quantification of (18)F-FLT human brain PET for the assessment of tumour proliferation in patients with high-grade glioma.. Eur J Nucl Med Mol Imaging.

[14] Zanotti-Fregonara P, Zoghbi SS, Liow JS, Luong E, Boellaard R (2010). Kinetic analysis in human brain of [(11)C](R)-rolipram, a positron emission tomographic radioligand to image phosphodiesterase 4: A retest study and use of an image-derived input function.. Neuroimage.

[15] Duman RS, Heninger GR, Nestler EJ (1997). A molecular and cellular theory of depression.. Arch Gen Psychiatry.

[16] Nestler EJ, Aghajanian GK (1997). Molecular and cellular basis of addiction.. Science.

[17] Briard E, Zoghbi SS, Imaizumi M, Gourley JP, Shetty HU (2008). Synthesis and evaluation in monkey of two sensitive 11C-labeled aryloxyanilide ligands for imaging brain peripheral benzodiazepine receptors in vivo.. J Med Chem.

[18] Fujita M, Imaizumi M, Zoghbi SS, Fujimura Y, Farris AG (2008). Kinetic analysis in healthy humans of a novel positron emission tomography radioligand to image the peripheral benzodiazepine receptor, a potential biomarker for inflammation.. Neuroimage.

[19] Carson RE, Barker WC, Liow J-S, Yao R, Thada S (2004). List-Mode Reconstruction for the HRRT.. J Nucl Med.

[20] Bloomfield PM, Spinks TJ, Reed J, Schnorr L, Westrip AM (2003). The design and implementation of a motion correction scheme for neurological PET.. Phys Med Biol.

[21] Fujita M, Zoghbi SS, Crescenzo MS, Hong J, Musachio JL (2005). Quantification of brain phosphodiesterase 4 in rat with (*R*)-[^11^C]rolipram-PET.. Neuro Image.

[22] Zoghbi SS, Shetty HU, Ichise M, Fujita M, Imaizumi M (2006). PET imaging of the dopamine transporter with ^18^F-FECNT: a polar radiometabolite confounds brain radioligand measurements.. J Nucl Med.

[23] Zubal IG, Harrell CR, Smith EO, Rattner Z, Gindi G (1994). Computerized three-dimensional segmented human anatomy.. Med Phys.

[24] Comtat C, Kinahan P, Defrise M, Michel C, Townsend D (1999). Simulating whole-body PET scanning with rapid analytical methods..

[25] Dusch E, Comtat C, Trebossen R (2009). Simulation-based evaluation of OSEM reconstruction bias on low activity PET data for the HRRT scanner.. IEEE Nuclear Science Symposium Conference Record (NSS/MIC) Oct. 24–Nov..

[26] Comtat C, Sureau F, Sibomana M, Hong I, Sjoholm N (2008). Image based resolution modeling for the HRRT OSEM reconstructions software.. IEEE Nucl Sci Symp Conf Rec.

[27] Mourik JE, Lubberink M, Schuitemaker A, Tolboom N, van Berckel BN (2009). Image-derived input functions for PET brain studies.. Eur J Nucl Med Mol Imaging.

[28] Naganawa M, Kimura Y, Nariai T, Ishii K, Oda K (2005). Omission of serial arterial blood sampling in neuroreceptor imaging with independent component analysis.. Neuroimage.

[29] Terry GE, Liow JS, Zoghbi SS, Hirvonen J, Farris AG (2009). Quantitation of cannabinoid CB1 receptors in healthy human brain using positron emission tomography and an inverse agonist radioligand.. Neuroimage.

[30] Terry GE, Hirvonen J, Liow JS, Zoghbi SS, Gladding R (2010). Imaging and quantitation of cannabinoid CB1 receptors in human and monkey brains using (18)F-labeled inverse agonist radioligands.. J Nucl Med.

[31] Brown AK, George DT, Fujita M, Liow J-S, Ichise M (2007). [^11^C]DASB imaging of serotonin transporters in patients with alcoholism.. Alcoholism: Clin Exp Res.

[32] Brown AK, Kimura Y, Zoghbi SS, Simeon FG, Liow JS (2008). Metabotropic glutamate subtype 5 receptors are quantified in the human brain with a novel radioligand for PET.. J Nucl Med.

[33] Litton JE (1997). Input function in PET brain studies using MR-defined arteries.. J Comput Assist Tomogr.

[34] Bodvarsson B, Hansen LK, Svarer C, Knudsen GM (2007). NMF on positron emission tomography..

[35] Lee DD, Seung HS (1999). Learning the parts of objects by non-negative matrix factorization.. Nature.

[36] Zanotti-Fregonara P, Maroy R, Sureau F, Comtat C, Jan S (2007). Noninvasive quantification of the cerebral metabolic rate of 18F-FDG in dynamic brain PET studies using an image-derived input function.. European Journal of Nuclear Medicine and Molecular Imaging.

[37] Rousset OG, Ma Y, Evans AC (1998). Correction for partial volume effects in PET: principle and validation.. J Nucl Med.

[38] Kimura Y, Ishii K, Fukumitsu N, Oda K, Sasaki T (2004). Quantitative analysis of adenosine A1 receptors in human brain using positron emission tomography and [1-methyl-11C]8-dicyclopropylmethyl-1-methyl-3-propylxanthine.. Nucl Med Biol.

